# Cemetery waste as a substream of municipal waste: research and structure of the selective waste collection in Poland

**DOI:** 10.1007/s11356-021-16351-7

**Published:** 2021-09-09

**Authors:** Anna Janda, Tadeusz Marcinkowski

**Affiliations:** Faculty of Environmental Engineering, Wroclaw University of Science and Technology, Wybrzeże Wyspiańskiego 27, 50-370 Wrocław, Poland

**Keywords:** Municipal waste, Cemetery waste, Waste management, Selective collection, Recycling, Zero-waste

## Abstract

Cemetery waste (CW) constitutes one of the streams of municipal waste (MW). Based on the available data, it can be said that it accounts for about 1% of the mass of MW being generated in Poland. CW management should be carried out selectively. It is estimated that the level of CW selective collection is lower than the average level achieved for MW. This paper presents some solutions concerning the selective collection of CW implemented in selected municipal and denominational cemeteries in Poland. Based on research conducted, the levels of proper separation of glass and biodegradable waste in containers for their selective collection were determined, accordingly as >85% and >80%. Due to the contamination with paraffin residues, the purity of the selectively collected plastic fraction was lower than 20%. The human factor was recognized as the most significant determining efficiency of the selective collection of CW. Therefore, further education of society, as well as promoting good practices in the field of CW management, should be regarded as desirable.

**Supplementary Information:**

The online version contains supplementary material available at 10.1007/s11356-021-16351-7.

## Introduction

Waste management is one of the greatest challenges for environmental protection in the modern world. Negative impacts of waste on the quality of the environment and human life and health, as well as an annually growing mass of generated waste forces comprehensive waste management starting from the stage of waste generation, through its treatment, ending up with its final disposal.

**Fig. 1:**
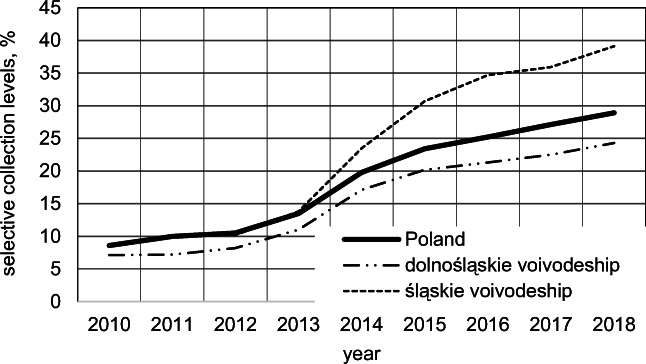
Levels of MW selective collection achieved in Poland (total) and selected voivodeships over the period 2010–2018 Office (file:///C:/Users/Administrator/Downloads/powierzchnia_i_ludnosc_w_przekroju_terytorialnym_w_2021_roku.pdf; Area and population in the territorial profile in 2021, Statistics Poland, Warsaw 2021)

Municipal waste (MW) management for member states of the European Union is based on directives, mainly on Directive 2008/98/WE on waste and repealing certain Directives ( 2008), implemented into national legislation. For Poland, the most important regulations governing procedures in waste management are the Act of Waste ( 2020) and The Communal Cleanliness and Order Maintenance Act (2020) together with the implementing acts. Currently, the main goal of MW management is to achieve required levels of recycling and preparation for reuse (i.e., min. 20% of the mass of MW generated in 2021, min. 25% in 2022, min. 35% in 2023, min. 45% in 2024, min. 55% in 2025, […], min 65% in 2035, and in each subsequent year), as well as reduction of waste directed to landfilling (max. 10% of generated municipal waste in 2030). Both of these objectives are to be accomplished by a steady increase in the levels of selective collection of MW and its processing in mechanical-biological facilities. The levels of selective waste collection in Poland in the years 2010–2018 were presented in Fig. 1. The figure also presents data for two selected voivodeships in order to compare achieved levels of selective waste collection for smaller areas of the country.

As the figure shows, the levels of selective collection of MW achieved in Poland were growing systematically over the period 2010–2018. The most dynamic changes were observed in the years 2012–2015. The increase in the selective waste collection on the national level in the years 2015–2018 was not more than 2% per year. The observed trend, as well as the level of selective collection of MW achieved in the year 2018, allows estimating the level of selective collection of MW achievable in the year 2020, not exceeding 35%. Data presented in Fig. 1 for dolnośląskie and śląskie voivodeships (south Poland) shows that the differences between the levels of selective collection achieved in various regions can be as high as a dozen or so percent.

To fulfill regulatory requirements concerning required levels of recycling (20% of generated MW in the year 2021), the remaining part of raw materials should be separated from the stream of mixed waste. This process is being performed in facilities designed for mechanical and biological treatment (MBT) during a staged separation, mainly of a granulometric fraction of a grain size >100 mm. According to reference literature, in the year 2017, the number of active MBT facilities in Poland amounted to 127, with an overall capacity exceeding the total mass of generated mixed MW (den Boer and Jȩdrczak 2017). MBT facilities also perform polishing of selected fractions of selectively collected waste (Połomka and Jędrczak 2019). Erroneous inlets, i.e., improperly collected material fractions, may constitute from 10% up to as much as 30% of the mass of the selectively collected waste. In selectively collected biowaste, the greatest part of pollutants is a fine fraction of inert waste (Alessi et al. 2020) and plastics (Hansen et al. 2007). In plastics, dominating pollutants are paper, cardboard, and glass. According to the available literature, about 30% of separated pollutants may be subjected to recycling (Malinowski et al. 2018).

The dominant source of MW are households. Under The Act of Waste, also wastes having composition or characteristics similar to household waste, e.g., cemetery waste (CW), defined as a subgroup of waste from gardens and parks (Code 20 01), are classified as MW. CW has two main sources of origin (Janda and Marcinkowski 2019). Their dominant source is used decorative elements of graves. In the stream of such waste, some fractions can be distinguished: glass (from memory lights, colorless, and colored), plastics (plastic grave lights, burnt paraffin refills, flower pots, packaging waste), and biodegradable waste (cut flowers, bouquets, and funeral wreaths). The second source of CW is conservating and repairing activities kept in cemeteries. As a result of clearing and arranging cemetery objects, mainly biodegradable waste is generated (grass, branches), as well as inert waste (soil, sand, aggregates, stones). Also, others than those listed waste may be generated, e.g., small amounts of metals, cardboard, textiles, and hazardous waste.

As a part of MW, CW should be collected selectively. In practice, selective separation of dominative material fractions, i.e., plastics, glass and biodegradable waste, is entirely reasonable for cemeteries. The remaining, trace fraction of waste should be deposited in containers for mixed or non-degradable waste. The person in charge of organizing waste collection is the cemetery administrator. For municipal objects, subordinated to the authorities of the commune, usually a board of cemeteries is created. For example, in Wrocław (*Poland, dolnośląskie voivodeship, population of 650,000 inhabitants*), such an organization governs six municipal cemeteries in the City (Marcinkowski and Janda 2019). In the case of denominational cemeteries, oversight is exercised by a religious community. As a result, the organization of selective CW collection systems in Polish agglomerations is very diverse.

Problems of collecting and neutralizing CW as a subgroup of MW have not been discussed in specialist literature. From the perspective of increased demands in the field of MW management, there is a need of undertaking integrated actions concerning all groups of MW, including CW. Only such actions will allow us to achieve the required levels of recycling and the reduction in the mass of waste directed to landfills.

This article is intended to present the current state of CW management in selected municipal and denominational cemeteries in Poland. Among others, accepted solutions of selective waste collection will be discussed. Based on balance research, the annual mass of generated MW was estimated, as well as changes in the stream of CW on an annual cycle. Based on earlier literature data and the research conducted, the levels of proper separation of glass and biodegradable fractions were calculated.

## The current state of CW management in Poland

### The amount of generated CW and its fluctuations in an annual cycle

Given the fact that CW belongs to the subgroup of waste from gardens and parks, its quantitative record is not kept. As can be found in the literature (Jaworska-Szott and Marcinkowski 2014), CW constitutes about 1% of the overall stream of MW. Detailed research for Wrocław during the period from May 2013 to April 2014 showed that for six municipal cemeteries, the total mass of CW generated amounted to 2.3·10^6^ kg per year, which was slightly above 1% of the total mass of MW generated in Wrocław. Due to individual management of each of the denominational cemeteries and resulting difficulties in obtaining reliable balance sheet data, the denominational cemeteries were not taken into account in the research. Their total area is about five times smaller than the area of municipal cemeteries, therefore their omission does not generate any significant error. A similar correlation between the mass of CW and the total mass of generated MW was observed in research conducted in Brno (*the Czech Republic, 405,000 inhabitants*) (Stejskal 2011).

**Fig. 2:**
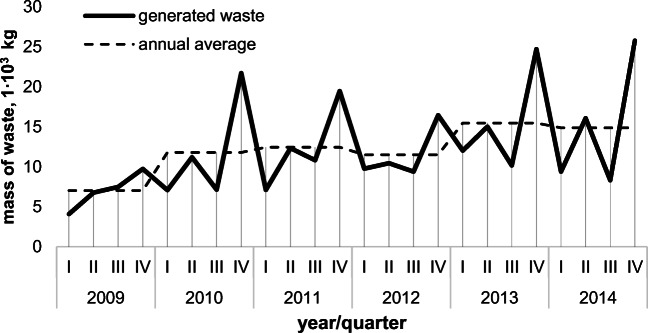
Variability of the mass of CW generated in the municipal cemetery in Wodzisław Śląski (śląskie voivodeship, Poland) over the period 2009–2014 (Bobrzyk, 2015; unpublished)

As a subgroup of MW, CW is characterized by variability in annual cycles. For MW, seasonal variations in the mass and composition of generated waste are caused by such factors as the type of residential development, heating system, and other factors (den Boer et al. 2010; Denafas et al. 2014). A study conducted in the years 2009–2014 in the municipal cemetery in Wodzisław Śląski (*Poland, Silesian Voivodeship, 50,100 inhabitants*) showed significant fluctuations in the size of CW stream in an annual cycle (Bobrzyk, 2015; unpublished). Research results achieved allowed to identify some repeating scheme of changes in the volume of CW in an annual cycle and was presented in Fig. 2.

**Fig. 3:**
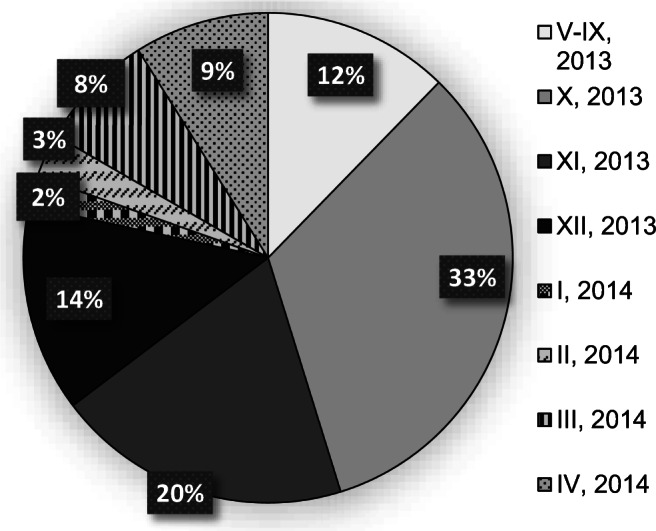
Percentage distribution of the mass of CW generated in particular months, based on research conducted in Wrocław (dolnośląskie voivodeship, Poland)

According to the information provided in Fig. 2, the mass of CW emerging in particular periods of the year shows clearly visible differentiation. For all the years under consideration, similar regularities can be found: the greatest amount of waste was generated in the last quarter of the year, the smallest in the third or the first quarter. The second quarter is also characterized by an increased stream of CW, but not exceeding the annual average. The identical trend was observed in research conducted for 6 municipal cemeteries in Wrocław in the season 2013/2014 (Jaworska-Szott and Marcinkowski 2014). The results are shown in Fig. 3.

Analyzing the results of the research presented in Fig. 3. We can conclude that more than 60% of the annual mass of CW was generated in the fourth quarter of the year. The increased stream of waste was also observed in March and April. For the period from May to September, the mass of waste generated was so small that no regular collection was performed in this period. Cumulated mass of waste generated during these months amounted to 12.3% of total mass (2.5% per month on the average).

Celebrations of Catholic holidays should be enumerated as a principal factor affecting changes in the CW stream in an annual cycle. During the period of Easter (March/April), All Saints Day (11.01) and All Souls’ Day (11.02), and at Christmas Holiday (12.25/26), the frequency of visits at the graves of loved ones grows significantly. Graves are then cleaned and decorated, mainly with flowers and memory lights. Finally, these objects are converted into a stream of waste, and cemetery users want to get rid of them quickly, easily, and “on-site.” Due to this, in the autumn (4th quarter) and spring (2nd quarter), the amount of generated CW is greater. Extremely high amounts are observed for the period from October to December when two important Catholic celebrations occur in a relatively short period.

Between the holiday periods mentioned, the stream of CW decreases. Waste generated in the first months of the year is mainly burnt grave lights and wilted flowers remaining after the November and December celebrations. In summer, despite the increased frequency of seasonal ordering actions in the cemetery, the mass of generated waste has the lowest value. This is due to partial decomposition of organic mass in waste, as well as evaporation of water already at the stage of its storage. In the research on samples of mixed waste from municipal cemeteries in Brno, it was found that the storage of waste in the months from April to July allowed the mass of waste to be reduced by about 40% (Stejskal 2014). High temperature during spring and summer months increases the intensity of decomposition processes in organic matter, as well as evaporation of water. Due to this, green waste generated by ordering works in cemeteries does not increase significantly the waste stream.

Variability of the volume of the CW stream as a function of time is the most important difficulty in the collection system for this waste. The levels of selective CW collection and its efficiency depend mainly on the number of containers for gathering particular waste fractions and on their location within a cemetery. Containers should be located close to the main traffic routes and close to the entrance gates of the cemetery. The principle of easy accessibility forces the use of a large number of containers. In winter and summer seasons, when the amount of generated waste has the lowest values, a large number of partially filled containers makes the process of waste removal quite difficult. For big cemeteries, it is possible to periodically reduce the number of containers in use. It was observed that in the vast majority of facilities, the frequency of waste removal during the periods between main celebrations is significantly reduced.

### Organization of the selective collection system

As it was mentioned in the introduction, accordance with Act of Waste and The Communal Cleanliness and Order Maintenance Act, CW should be collected selectively. Due to the composition and nature of this waste, it is justified to separate the fractions of glass, plastics and metals, and biodegradable waste. According to the Communal Cleanliness and Order Maintenance Act (2019), a selective waste collection should be performed since the year 2013. The information about the necessity of selective waste collection should be placed, e.g., on entrance gates of the cemetery and in the terms of use of the facility. After being collected, the CW stream is directed along with the remaining stream of selectively collected MW to MBT facilities.

Due to different authorities governing municipal and denominational cemeteries, the structure of CW collection in Poland differs widely. For example, in the Osobowice cemetery, the greatest municipal cemetery in Wrocław with area of 0.53 km^2^, each of the fractions mentioned earlier is being separated since the year 2013 (Janda and Marcinkowski 2019). However, various sources inform that standard containers designed for mixed waste still are the dominant type of containers in the majority of cemeteries. Due to this, achieved levels of CW selective collection are significantly lower than those required by Polish and UE regulations. Cubic capacity data of containers for CW selective collection in selected cemeteries in Poland are presented in Table 1.

**Table 1:** Containers applied to CW collection, including selective collection (sc) of fractions of plastics (pl), glass (gl), biodegradable waste (b), and green waste (gr) in selected cemeteries in Poland

Cemetery, (area, km^2^)	Year	Capacity		Containers for sc	sc fractions	[Ref.]*
		Total	For sc			
		m^3^	m^3^	%		
Wodzisław Śl. (0.04)	2010	25.5	4.5	17.6	pl,gl	(Bobrzyk, 2015)
	2014	44.0	9.0	20.5	pl,gl	
Osobowice (0.53)	2013	318.9	79.1	24.8	pl, gl, b	(Ziemiński, 2018)
	2018	408.1	200.1	49.0	pl, gl, b, gr	
St. Lawrence (0.01)	2015	33.0	0.0	0.0	–	(Chmielewska, 2015)
	2018	32.2	2.5	7.9	pl, gl, b	(Prędkiewicz, 2018)
Świdnica pd. (0.08)	2017	75.6	17.6	23.3	pl, gl, b	(Orzechowski, 2017)
st. Michael (< 0.01)	2018	2.2	0.5	22.7	gl	(Prędkiewicz, 2018)

^*^Data based on master’s theses carried out at the Wrocław University of Science and Technology in 2015–2018

As can be seen in Table 1, the organization of the CW selective collection system is an individual task for each facility. At most cemeteries, all material fractions, i.e., plastics, glass, and biodegradable waste, are separated. The largest percentage of containers designed for the selective collection, almost 50% of the total cubic volume of containers, was recorded in the Osobowice cemetery. This is also the only cemetery having separate containers for green waste. For each of the cemeteries analyzed, containers for mixed waste are the dominant type of waste containers. The comparison of data over the years for three cemeteries show that the number of containers for selective collection grows systematically. The growing volume of containers also shows an increasing volume of the stream of CW generated.

### Selective CW collection efficiency

Due to the lack of detailed records on the mass of generated CW, it is impossible to evaluate the current levels of its selective collection. Research on the municipal cemetery in Wodzisław Śl. in the years 2010–2014 showed that the levels of CW selective collection amounted from 8.0 to 15.5% and did not differ significantly from the results for MW collection in Poland and Silesian voivodeship (Fig. 1). The exception was the year 2014 when the levels of CW selective collection were lower by, respectively, 4.3% and 8.0%, in relation to the country and the voivodeship (Bobrzyk, 2015, unpublished). Research on municipal cemeteries in Wrocław in the year 2014 showed the level of CW selective collection equal to 15%, i.e., lower by, respectively, 4.8% and 2.1%, in relation to the country and the dolnośląskie voivodeship (Jaworska-Szott and Marcinkowski 2014).

Apart from the achieved levels of selective waste collection, an important parameter is also the efficiency of selective collection. This term refers to the correctness of the separation of material fractions and determines the purity of the obtained material fractions and the share of erroneous inlets. Research carried out so far on the efficiency of a selective collection in two facilities in Wrocław: Osobowice cemetery and St. Lawrence cemetery showed that the level of proper separation of basic material fractions ranged between about 80 and 92% (Janda and Marcinkowski 2019). Analysis of the content of containers for non-degradable waste showed that the percentage of a green fraction (i.e., erratic inlets) can amount to as much as 40% of their total load. The most efficiently separated fraction is glass, and its important contaminant is plastics. A similar trend was observed in containers for plastics, where the glass was a significant contaminant. This is due to the multi-material structure of grave lights that usually consist of a glass housing, plus metal and plastic elements (a base, a cap, and a refill).

The percentage of green fraction in containers for biodegradable waste was from 80 to 85% and its most important part was a green coniferous fraction. As in the case of grave lights, bouquets are also composed of more than one material. Dumping of this type of waste without prior dismantling results in decreased efficiency of selective collection and decreased vulnerability of such a waste to biological stabilization processes.

Results of the research presented above concern proper separation of material fractions in spring and summer periods, when the quantity of generated CW is the smallest. These results cannot be related to selective waste collection in the periods of Catholic holidays when the mass of generated waste is several times larger. Because of this, complementary studies were carried out, including identification of proper separation of selectively collected fractions of CW in the periods before and after Christmas and Easter. The course of the study and its results are presented below.

## Research

In the research, the effect of changes in the volume of CW stream on the efficiency of proper separation of the selected material fraction was determined. The research was intended to determine the percentages of particular material fractions of waste collected in containers (see Table 2). For the purpose of the study, on each cemetery few containers intended for the collection of glass, plastics, biodegradable, and mixed waste were selected. The containers for the study were chosen mostly by their localization so that the samples were as representative as possible.

**Table 2:** The quantity and cubic volume of containers for CW collection in the cemeteries in Świdnica, in the years 2016 and 2017 (Orzechowski, 2017; unpublished)

Type of the waste container	Northern cemetery			Southern cemetery		
	Volume, m^3^	Number, pcs.	Total volume, m^3^	Volume, m^3^	Number, pcs.	Total volume, m^3^
Plastics	2.2	2	4.4	2.2	4	8.8
Glass	2.2	2	4.4	2.2	4	8.8
Biodegradable waste	–	4	–	–	4	–
Mixed waste	2.2	14	30.8	5.5	1	5.5
	7.5	3	22.5	7.5	7	52.5
**Total**	**–**	**25**	**62.1**	**–**	**20**	**75.6**

### Research area

The research was carried out on real waste samples from two municipal cemeteries in Świdnica (*south Poland, dolnośląskie voivodeship, 60,000 inhabitants*). The cemeteries were located in the suburbs of the city, in its northern (area of 0.04 km^2^, 6300 graves) and southern (area of 0.08 km^2^, 6500 graves) parts of the city. The size of the cemeteries covered by the study allows them to be classified as medium-sized objects, within the size range between small denominational cemeteries and large, metropolitan municipal cemeteries (see Table 1).

Świdnica was selected as the research area due to the short distance from Wrocław (about 60 km) and the similar number of inhabitants to Wodzisław Śląski, where the first research on CW stream were conducted. The decisive factor in choosing the location of the research was also obtaining approval for their realization granted by the city government. Due to the fact that Świdnica belongs to the group of 10% of the largest cities in Poland (https://bdl.stat.gov.pl, 2021) and taking into account the size of municipal cemeteries, the selected research area can be considered as a reference unit for Poland.

### Research methodology

In the first stage of the research, the number and volume of containers for selective waste collection in both municipal cemeteries in Świdnica were determined.

In the next stage of the work, a morphological content analysis of waste gathered in selected containers was carried out. For that purpose, waste from each container was divided into basic material fractions. In the study, the following waste separation was adopted: the fractions dominating in the CW stream, i.e., glass, plastics and metals, biodegradable waste, inert waste (soil, sand, stones, ceramics), and other waste. “Other waste” was defined as all fractions that are not included in the selective waste collection system in the cemetery, i.e., paper and cardboard, multi-material waste, textiles, and hazardous waste. In the case of the containers for biodegradable waste, all non-biodegradable fractions of waste were defined as “other waste.”

In the next step, mass analysis of separated material fractions was performed. Measurements were made using a hook scale with a load capacity of up to 50 kg and accuracy of ± 0.001 kg. Masses of particular fractions were then converted to percentages in relation to the total mass of the waste. On the basis of the obtained measurement data, the share of erroneous inlets in containers for selective CW collection was determined. To eliminate the effect of atmospheric precipitation on the mass of biodegradable waste deposited outdoors, the atmospheric conditions during the study were also recorded. Because of the lack of heavy rainfall in a periods shorter than 3 days from the dates of measurements, the effect of atmospheric conditions on the mass of the fractions mentioned above was eliminated.

The analyses described were carried out shortly before Christmas (from 12 to 21 December 2016) and Easter (from 10 to 13 April 2017) and shortly after those holidays (from 7 to 10 January 2017 and from 19 to 24 April 2017). To compare the results with the efficiency of selective collection during a period of small waste accumulation, similar test were performer in a period between holidays (from 13 to 15 March 2017).

### Results

In both municipal cemeteries in Świdnica, the selective waste collection was implemented in the fourth quarter of the year 2016, and during the period of the study, it included the separation of the fractions of glass, plastics and metals, and biodegradable waste. Detailed data on the quantity and volume of containers for particular material fractions in the period of 2016–2017 are summarized in Table 2.

The total cubic volume of waste containers in the northern cemetery was greater than in the southern cemetery. This is due to the fact that new graves are no longer created in the northern cemetery, burials are rare, and some old graves are not visited. This translates into a smaller annual stream of MW in the southern cemetery. Containers for mixed waste were the predominant container type in both cemeteries. In the southern cemetery, the containers for selective collection of glass and plastics accounted for 23.3% of the total volume of waste containers. In the northern cemetery, the share of these containers accounted for 14.2%. In both cemeteries, biodegradable waste was collected in fenced boxes, or “bulk” by the containers for mixed waste. It was therefore impossible to determine its exact volume.

**Fig. 4:**
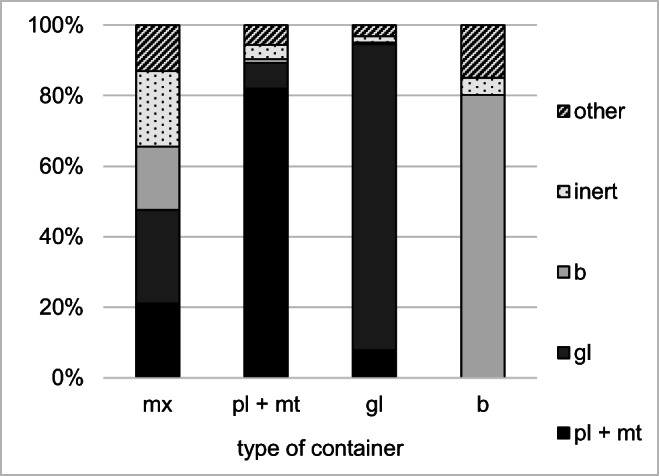
Efficiency of CW selective collection in cemeteries in Świdnica in March 2017 in containers for mixed waste (mx), plastic and metals (pl + mt), glass (gl), and biodegradable (b) waste fractions (Orzechowski, 2017; unpublished)

To determine the changes in efficiency of CW selective collection in the holiday periods, it was necessary to examine the content of containers in the period between holidays, as a reference point. The averaged results of the tests of composition and morphology of the content of the containers for waste collection in both cemeteries in Świdnica in the period between holidays were summarized in Fig. 4. During sampling, the containers were filled in not more than 60%.

As shown in Fig. 4, the efficiency of proper separation of material fractions in the cemeteries in Świdnica in the period between holidays, for the containers collecting glass, plastics, and biodegradable waste, amounted to, respectively, 87%, 82%, and 85%. These values are lower than those obtained in the case of the research performed in the cemeteries in Wrocław.

The dominant contaminants of the glass fraction were plastics (7.8%) and vice versa: plastics were contaminated mostly by glass (7.2%). The same trend was noted in the study performed in cemeteries in Wrocław. In the case of the containers for biodegradable waste, the green fraction amounted to 80% of the total mass of the waste. As much as 15% of the mass of waste gathered in these containers were “other waste;” these were mostly plastics and other packaging waste. Due to the specific nature of green waste, the inert waste fraction was always present in the containers for its collection. Soil attached to the plant root system, when thrown into the container, is dried and separated from plants; therefore, its share should not be concerned as a contaminant of the green fraction. Because of this, the value of the effectiveness of the selective collection of biodegradable waste in the period between holidays was assumed at 85%. In the containers for the mixed waste collection, each of the main material fraction was from 21 to 26% of the total mas of waste, and other fractions amounted to about 13% of the total mass.

**Fig. 5:**
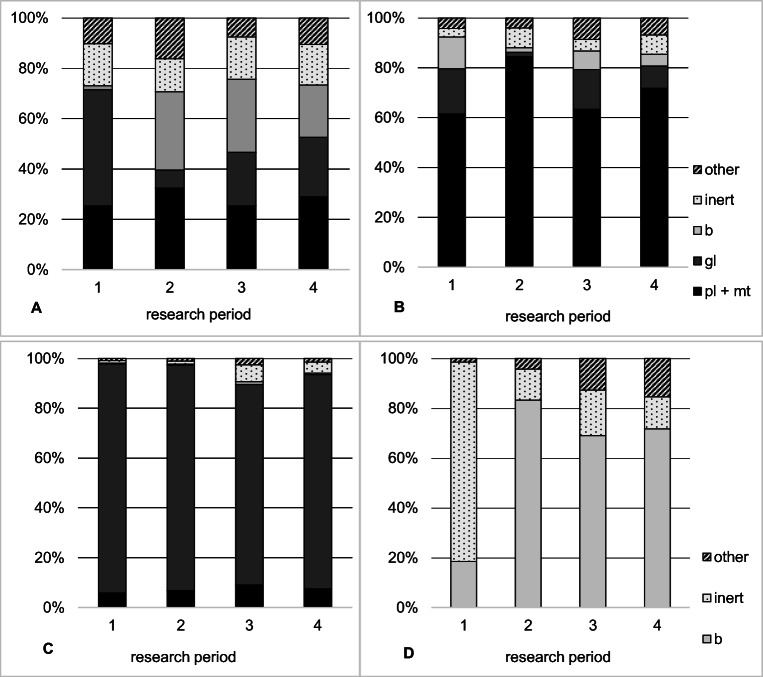
Percentage of selected material fractions in the periods before Christmas (1) and before Easter (3), and after those holidays (2, 4), in the containers collecting: **A** mixed waste, **B** plastics and metals (pl + mt), C) glass (gl), and **D** biodegradable waste (b) (Orzechowski, 2017; unpublished)

The averaged results of the research on the material composition of waste collected in the cemeteries in Świdnica in the periods before and after Christmas and Easter are summarized in Fig. 5. On the days of sampling, the containers filling levels were from 50 to 80%.

As shown in Fig. 5A, glass collected in the containers for mixed waste accounted for from 7% to as much as 50% of the mass of waste, which was far from the values recorded in the period between holidays (26.5%). In the December research series, the percentage of green fraction in the containers for mixed waste was practically negligible. For the other months, this percentage was higher than in the period between holidays. The shares of inert waste and plastics in the containers for mixed waste remained at a similar level in all analyzed months of the year. In all periods of study, the filling level in the containers for selective collection of all material fractions did not exceed 70%.

The test results compiled on the 5C chart confirm that the glass fraction is the most efficiently separated material fraction, also in the period of high waste accumulation. In December and January, glass represented more than 90% of mass content in the containers intended for the collection of this material fraction. In the periods before and after Easter, the efficiency of the selective collection was lower and accounted for, respectively, 81% and 86%. Plastic waste was invariably the largest contamination of the glass fraction by several percents; this was mainly burnt refills from paraffin grave candles. In April, an increased percentage of the inert waste fraction was observed. This could be the result of the incorrect collection of ceramic waste in containers intended for collecting glass.

Figure 5D shows the share of different types of waste in containers for the selective collection of biodegradable waste. Considering green waste and inert waste as correctly collected fractions, the efficiency of their selective collection can be evaluated as 85 to 99%, which is a better result than it was obtained during the periods between holidays. Around Easter, an increase in the share of the remaining fractions, mostly plastics, was observed. Before Christmas, the dominant fraction in the containers was the inert fraction, and this was due, among other things, to the removal of the flowers planted directly in the graves after autumn. The soil combined with the root systems has a higher mass than the dry parts of green plants, thus resulting in a mass fraction of which was higher than the proportion of green waste.

As shown in Fig. 5B, the efficiency of selective collection of plastics in the periods of holidays was in three cases lower than in the periods between holidays, and it amounted to from 62 to 84%. The results obtained in each of the research periods were lower than those obtained for cemeteries in Wrocław. In the holiday period, in containers for plastics collection, some increase in the percentage of the green fraction was noted. This fraction constituted a negligible mass share when the waste accumulation level was low. Also, the share of the “other” waste was higher and this was mainly cardboard packaging waste.

**Fig. 6:**
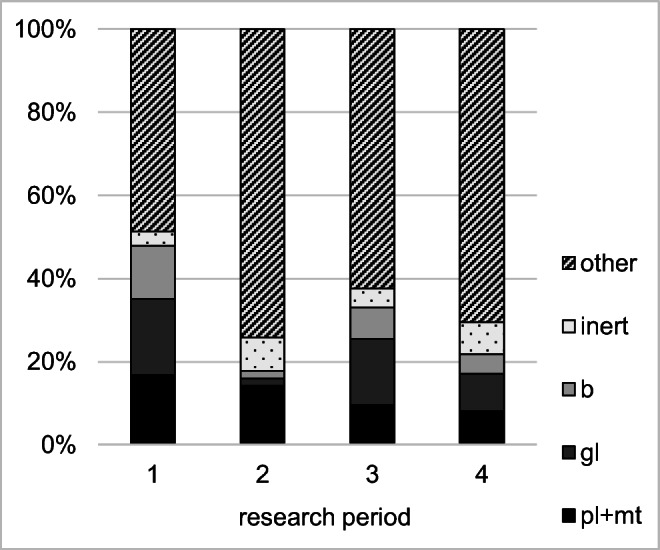
Percentage of selected material fractions in the periods before Christmas (1) and before Easter (3), and after those holidays (2, 4), in the containers for selective plastic collection (b, biodegradable waste; gl, glass; pl+mt, plastic and metals)

The data presented in Fig. 5B include that all plastic waste are a correctly deposited material fraction. However, it must be remembered that highly contaminated plastic waste impedes the recycling process, so it should not be collected selectively. This problem mainly concerns greasy waste, which requires washing with solvents and/or hot water during the recycling processes. This is associated with high costs and generation a wastewater stream with a high load of organic compounds (Altieri et al. 2021). Highly contaminated paraffin refills are the dominant type of plastic waste generated on cemeteries. Because of large amounts of unburned paraffin residues, they should be disposed of in mixed waste containers. However, research carried out in Świdnica has shown that used refills may constitute up to 93% of the mass of plastics deposited in containers for their selective collection. Detailed data are presented in Fig. 6.

Figure 6 presents the same data as Fig. 5B, but with a different definition of the correctly deposited plastic fraction. In Fig. 6, contaminated paraffin refills were defined as a pollution of plastic fraction and included in fraction “other.” In this situation, a purity of selectively collected plastic fraction is lower than 20%.

## Discussion

The test results obtained and presented above do not allow a clear indication of the impact of increasing the amount of CW on the degree of separation of basic material fractions. In the case of plastics, there was a reduction in the efficiency of their proper separation compared to the period between holidays by up to 20%. For the remaining fractions, the results obtained during the Christmas season are similar or slightly higher than during the periods between holidays. According to the above observations, it can be stated that the increased CW stream has no direct impact on the fluctuations in the efficiency of selective waste collection.

The test results obtained for the cemeteries in Świdnica significantly coincide with the results regarding the selective collection at cemeteries in Wrocław. On this basis, based on previous literature reports and the research results obtained, the overall efficiency of the correct separation of the selected material fractions of CW can be established.

The efficiency of selective glass waste collection is the highest and ranges from about 85 to 90%. This result is influenced, among others, by the higher specific gravity of glass compared to other material fractions of the waste. For this reason, glass constitutes such a high mass share in containers for its collection. The opposite can be observed for very light plastics. Contamination of the selectively separated plastic fraction with glass by several percent may in practice mean the incorrect placement of several glass candles in the container.

The efficiency of selective collection of the biodegradable fraction defined as the fraction of green and inert waste is higher than 80%. The proportion of the soil as an inseparable component of green waste may constitute several to even several dozen percent of the weight of this waste.

High contamination of collected plastics fraction generated in cemeteries prevents their material and/or chemical recycling. Removing paraffin residues is an expensive process, which makes recycling of plastics less profitable. However, this type of waste can be used in thermal energy recovery processes (Jeswani et al. 2021).

Analysis of the composition of waste in containers for the mixed fraction allows the identification of the main factors determining the efficiency of selective waste collection. Despite only partial filling of containers for selective waste collection, the high availability of containers for mixed fractions resulted in the accumulation of waste glass, plastic, and biodegradable fraction. This situation was most visible for the glass fraction, where for the same conditions of organization of the collection system and only partial filling of containers for selective glass collection, it constituted from 7 to 50% of the weight of deposited waste.

**Fig. 7:**
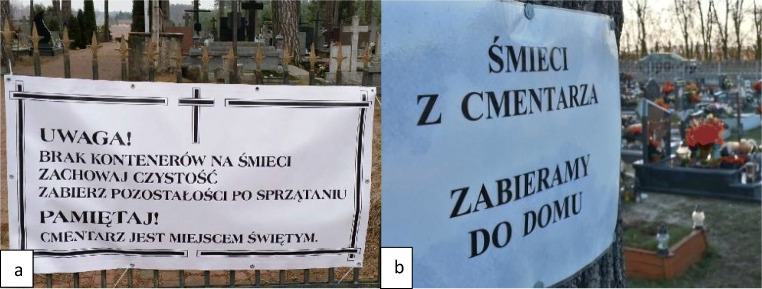
Announcements regarding the lack of collection of CW on the premises of denominational cemeteries in **a** Michałów (Poland, warmian-masurian voivodeship) “Attention, no garbage containers available. Keep clean. Take leftovers after cleaning” and **b** Kalinów (Poland, opolskie voivodeship) “garbage from the cemetery we take home”; (https://nto.pl/urzednicy-do-proboszcza-gdzie-sa-smieci-z-cmentarza/ar/4607121, https://plus.poranny.pl/michalowscy-przedsiebiorcy-musza-sami-podpisywac-umowy-na-odbior-smieci-podpisuja-i-sie-dziwia/ar/c1-14732368, https://plus.poranny.pl)

The increased CW stream is the result of an increase in the frequency of visits to cemeteries and it is the human factor that should be identified as directly affecting the efficiency of the separation of waste material fractions. Erroneous throws can be caused by insufficient knowledge of cemetery users about selective waste collection and/or failure to respect generally accepted principles especially in public places. CW is a specific stream of MW with characteristic properties that differ from the properties of waste generated in households. The multi-material structure of candles and wreaths as well as the high degree of contamination of some fractions (mainly glass and plastics) with paraffin impedes the correct separation of waste. Unawareness and lack of sufficient involvement on the part of visitors to cemeteries result in the formation of a dominant mixed waste stream and a heavily contaminated waste stream selectively collected treated in practice as a mixed waste stream. Rising prices of mixed waste collection sometimes result in drastic moves on the part of cemetery managers, consisting in the complete abandonment of waste collection at the cemetery (see Fig. 7). These types of activities usually concern small-scale denominational cemeteries and face considerable opposition from society. In Polish legislation, however, there is no clearly defined provision ordering the collection of MW at the place of its creation. However, it can be assumed that following the principle of proximity mentioned in the Act of Waste (2019), waste should be collected “at source.”

**Fig. 8:**
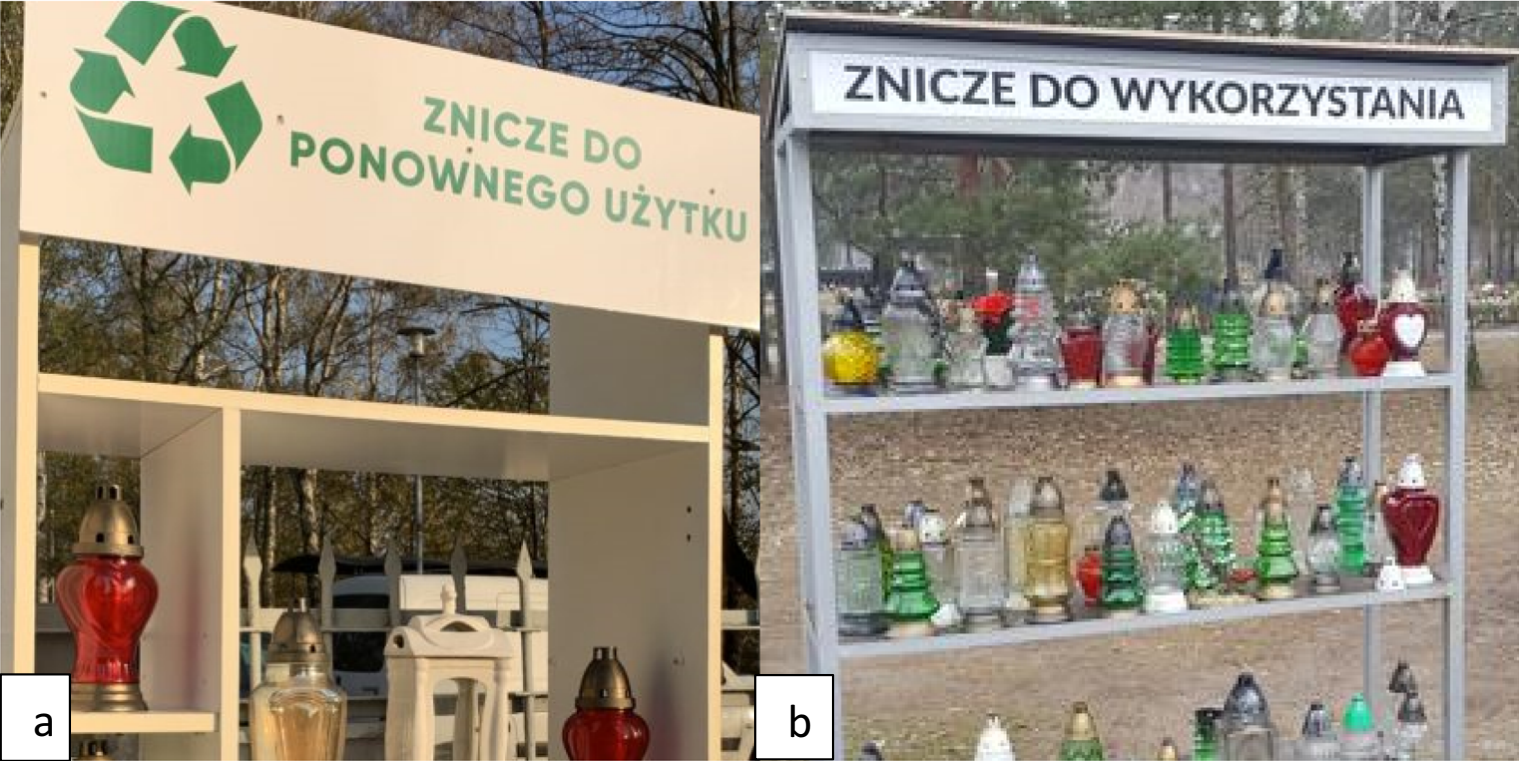
The “candles for reuse” initiative at municipal cemeteries in **a** Częstochowa (Poland, śląskie voivodeship) and **b** Bydgoszcz (Poland, kujawsko-pomorskie voivodeship); (https://zycieczestochowy.pl/drugie-zycie-zniczy/, https://www.se.pl/bydgoszcz/na-cmentarzu-w-bydgoszczy-pojawily-sie-polki-na-znicze-to-doskonaly-pomysl-audio-aa-brVC-1YNG-saro.html, https://www.se.pl/bydgoszcz)

The characteristic properties and composition of CW and the resulting problems with waste management induce both cemetery managers and the society to organize projects that promote compliance with the highest levels of the waste hierarchy, i.e., waste prevention and preparation for reuse. An example of good CW practice is appearing recently in the cemeteries, candles shelves promoting the idea of “Candles for reuse” (see Fig. 8). This action is part of the “zero-waste” trend, which is growing in popularity, i.e., actions leading to the reduction of generated waste to a minimum (Mesjasz-Lech 2019; Zotos et al. 2009). Another example of good practice in the CW economy is the separation of pots after flowering plants, carried out, e.g., at the denominational cemetery in Wysoka (Poland, 1000 inhabitants, opolskie voivodeship). The separated materials are handed over to local horticultural and fruit farms. The administrator of this cemetery also encourages parishioners, i.e., the main users of the cemetery, to purchase candles made of transparent glass (an attractive raw material for recycling) with the possibility of multiple uses by replacing used paraffin refills. These actions not only result in reducing the stream of CW generated but also increase public awareness in the aspect of the complexity of CW economics.

## Summary and conclusions

Cemeteries are a place of generation of municipal waste (MW), which according to EU and national law should be collected selectively. Despite the relatively small amount of cemetery waste (CW), the systematic tightening of requirements for the MW economy forces the implementation of the best waste management systems also in cemeteries. The organization of the CW selective collection system shall be implemented on a case-by-case basis by the administration of the facility concerned. Selectively separated material fractions should include at least: glass, plastics, and biodegradable waste.

Studies carried out in Poland and the Czech Republic have shown that CW represents approximately 1% of the total MW stream for the agglomerations studied. The amount of CW in the annual cycle is variable and increases in periods associated with the celebration of Catholic holidays. The smallest amounts of waste are generated in the winter and summer months. Lack of up-to-date balance data on the mass of generated CW makes it impossible to determine the achieved levels of selective collection. The results of research carried out at the municipal cemeteries in Wrocław and Wodzisław Śląski in the years 2010–2014 suggest that the obtained levels of selective CW collection are lower than the levels obtained for MW in Poland. To increase the level and efficiency of separate CW collection, it is necessary to gradually reduce the number of mixed waste containers in favor of increasing the number of containers for separate collection

The research carried out at Świdnica cemeteries did not allow us to define the impact of increasing the CW stream in the periods around holidays on the efficiency of the correct separation of selectively collected material fractions. Due to the convergence of the results obtained with the physical data on the collection of CW in Wrocław, the average proportion of false discards for the glass fraction and biodegradable waste was determined at a level corresponding to < 15% and < 20% respectively. The purity of selectively collected plastic fraction was lower than 20%, mainly because of high content of plastic refills contaminated with paraffin residues.

As in the case of MW from households, the human factor determines the effectiveness of selective CW collection the most. Too high level of incorrect throws in selectively collected CW fractions results in treating them as mixed waste. For this reason, it is necessary to inform cemetery users about the need for selective waste collection through messages located at the entrance gates to the cemetery area and directly on containers for the collection of waste. Enforcing compliance with the principles of selective CW collection at large municipal cemeteries (e.g., in Wrocław) is less effective than at cemeteries in smaller agglomerations (e.g., in Świdnica) and church denominational cemeteries.

The aspects of CW management presented in this publication prove the complexity of this issue. The low share of CW in the MW stream, its characteristic properties, and a high degree of contamination of selected material fractions induce reflection on the appropriateness of collecting CW selectively.

In our opinion, existing separate collection systems for CW could be limited only to the separation of glass, which despite being contaminated can be recycled. Selective collection of plastic fractions and biodegradable waste does not result in increasing the achieved recycling levels. The best way to manage plastics produced in cemeteries seems to be their energetic use. For this purpose, plastics could be separated from the mixed waste stream in the MBT facilities and directed to the refuse derived fuel stream (RDF). The fraction of biodegradable waste with a high share of inert waste and other pollutants (including plastics) should not be directed to organic recovery processes like composting. These waste stream should be rather directed to biological stabilization processes where the final product can be safely stored or to fermentation processes with biogas production.

However, the current state of knowledge regarding the properties and composition of CW is insufficient to accurately define the best ways to collect and further manage this waste. Therefore, it is necessary to continue balance and morphological research on CW streams.

## Supplementary Information

ESM 1 (DOCX 16 kb)

## Data Availability

The authors declare that all necessary data supporting the findings of this study are available within the article. The datasets from master’s thesis analyzed during the current study are not publicly available in accordance with the policy of the Wroclaw University of Science and Technology.
