# Supplementary material for: Cemetery waste as a substream of municipal waste: research and structure of the selective waste collection in Poland
Source: Environ Sci Pollut Res Int. 2021 Sep 9;29(7):9722–33. doi: 10.1007/s11356-021-16351-7 (PMC8783916; doi:10.1007/s11356-021-16351-7)
Supplement: Supplementary file 1 — (DOCX 16 kb) [file 11356_2021_16351_MOESM1_ESM.docx]

| **Lp.**  **List of changes**  Symbol „-„ means, that this part of text was added after the review process.  The line number relates to the changed text. | **Verse** | **old version** | **change** |
| --- | --- | --- | --- |
| 1. | 36 | effectiveness | levels |
| 2. | 36-38 | - | Figure also presents data for two selected voivodeships in order to compare achieved levels of selective waste collection for smaller areas of the country. |
| 3. | 45 | - | Per year |
| 4. | 49 | voivodeships | regions |
| 5. | 191-193 | - | Apart from the achieved levels of selective waste collection, an important parameter is also the efficiency of selective collection. This term refers to the correctness of the separation of material fractions and determines the purity of the obtained material fractions and the share of erroneous inlets. |
| 6. | 221 | - | south |
| 7. | 223-231 | - | The size of the cemeteries covered by the study allows them to be classified as medium-sized objects, within the size range between small denominational cemeteries and large, metropolitan municipal cemeteries (see table 1).  Świdnica was selected as the research area due to the short distance from Wrocław (about 60 km) and the similar number of inhabitants to Wodzisław Śląski, where the first research on CW stream were conducted. The decisive factor in choosing the location of the research was also obtaining approval for their realization granted by the city government. Due to the fact that Świdnica belongs to the group of 10% of the largest cities in Poland (Statistics Poland, 2021) and taking into account the size of municipal cemeteries, the selected research area can be considered as a reference unit for Poland. |
| 8. | 529-530 | - | file:///C:/Users/Administrator/Downloads/powierzchnia_i_ludnosc_w_przekroju_terytorialnym_w_2021_roku.pdf; Area and population in the territorial profile in 2021, Statistics Poland, Warsaw 2021. date of access 16.08.2021 |
